# From Growth Factors to Structure: PDGF and TGF‐β in Granulation Tissue Formation. A Literature Review

**DOI:** 10.1111/jcmm.70374

**Published:** 2025-06-11

**Authors:** Josiah Irma, Arief S. Kartasasmita, Angga Kartiwa, Irawati Irfani, Saraswati Anindita Rizki, Serena Onasis

**Affiliations:** ^1^ Doctoral Program in Medical Sciences, Faculty of Medicine Padjadjaran University Bandung West Java Indonesia; ^2^ Ophthalmology Department Pelita Harapan University Tangerang Banten Indonesia; ^3^ Department of Ophthalmology, Faculty of Medicine Padjajaran University Bandung West Java Indonesia; ^4^ Faculty of Medicine Pelita Harapan University Tangerang Banten Indonesia

**Keywords:** granulation tissue formation, PDGF, PDGF‐AA, PDGF‐AB, PDGF‐BB, TGFβ, wound healing

## Abstract

Platelet‐Derived Growth Factors (PDGFs) and Transforming Growth Factor β (TGFβ) are pivotal in orchestrating the complex wound healing process, particularly in granulation tissue formation. This review aims to comprehensively examine the roles of PDGF alongisde TGFβ in granulation tissue formation and their implications for abnormal wound healing. PDGFs, as homodimeric or heterodimeric combinations, such that PDGF‐AA, PDGF‐AB and PDGF‐BB stimulate fibroblast proliferation and extracellular matrix synthesis, which is crucial for tissue repair. TGFβ, with its three isoforms, influences granulation tissue through diverse functions, with TGFβ‐1 pivotal in fibrosis formation. Understanding their signalling pathways, notably PDGF's engagement with PDGF receptors and subsequent activation of cellular pathways, illuminates their roles in wound healing cascades. Excessive granulation, a complication of abnormal wound healing, involves dysregulated PDGF and TGFβ activity, leading to hypertrophic scar formation. Clinical management, particularly in ophthalmology, addresses excessive granulation's impact on procedures like endo‐dacryocystorhinostomy. Strategies employing steroid agents and Mitomycin‐C aim to mitigate ostium granulation. The potential use of PDGF receptor blockers, such as olaratumab, warrants further investigation for managing excessive granulation. In conclusion, PDGF and TGFβ emerge as critical regulators in granulation tissue formation, underscoring their significance in wound healing processes and offering avenues for therapeutic intervention.

## 1 Introduction

Multiple factors may influence the optimal wound healing process, including biochemical disruptions such as dysregulated growth factors. Platelet‐derived growth Factors (PDGFs) are biological signals that stimulate chemotaxis, mesenchymal cell proliferation and gene expression for monocytes‐macrophages. They play a vital role in multiple subsequent stages of wound healing—especially granulation tissue formation [1, 2, 3, 4]. PDGFs are disulfide‐linked homodimers or heterodimers forming a dimeric combination of PDGF‐AA, ‐BB and ‐AB which bind to two receptors of PDGFs, PDGFRα and PDGFRβ. Its impact on fibroblast proliferation, migration, activation and their influence on re‐epithelization, collagen deposition and angiogenesis are essential for active wound healing. However, PDGFs are also associated with chronic non‐healing wounds [1, 2, 3]. Aside from PDGF, Transforming Growth Factor β (TGFβ) is a significant growth factor in wound healing due to its effects on fibroblasts, with TGFβ being a dominant factor in this process [5, 6]. Dysregulated growth factor levels may result in excessive granulation tissue formation as a common complication of wound healing. Granulation tissue forms during the proliferative phase, where a dense network of blood vessels, fibroblasts and collagen fibres provide a scaffold for new tissue formation to facilitate healing. However, when the balance of this process is disrupted, hyper‐granulation can occur, obstructing reepithelization and prolonging wound closure. The open wound then creates a conducive environment for infection to occur and may further result in psychological distress, potential scar formation and delayed healing. Although many other factors such as Vascular Endothelial Growth Factor (VEGF) and Fibroblast Growth Factor (FGF) are involved in wound healing, PDGF and TGFβ are the two main significant factors, and cross‐talk between the two showed enhancement of TGFβ signalling, thus why this review aims to provide a comprehensive review of the specific functions of PDGF and TGFβ in the process of granulation tissue formation [7].

## 2 Discussion

### 2.1 An Overview of the Wound Healing: The Role of PDGF and TGFβ

Wound healing is a complex process consisting of 4 stages: haemostasis, inflammation, proliferation and remodelling. It is a biological response involving numerous cells, growth factor signals and cytokines for wound closure. A wound is created when any tissue injury disrupts epidermal layer integrity, hence creating a loss of protective function. Pathogen invasion and fluid loss may occur when this happens. Thus, abrupt initiation of the wound healing process is necessary to prevent infection. An uncomplicated wound may heal well with primary healing intention. However, any disruption during the process, such as infection, hypoxia, or immune dysfunction, may result in a secondary healing stage leading to chronic wounds, such as—excessive wound healing or chronic wound formation. Hence, an optimal wound needs organised interaction between cellular, humoral and molecular mechanisms, including PDGF‐AA and PDGF‐BB, which interplay a crucial role in wound formation, specifically granulation tissue formation [8].

Haemostasis occurs immediately after tissue injury to stop the bleeding and is followed by inflammation to seal the wound surface and remove necrotic tissue, debris and bacteria. Haemostasis involves local arteriolar and capillary vasoconstriction followed by vasodilation and increased vascular permeability [5]. Platelets and erythrocytes adhere to disrupted endothelium. The platelet then binds to exposed type IV and type V collagen. This activates platelets and causes the release of alpha granules consisting of PDGF, TGFβ, IGF‐1, Fibronectin, Fibrinogen, Thrombospondin, Von‐Willebrand Factor and Serotonin [5, 9]. Fibrinogen is cleaved into fibrin meshwork by thrombin, allowing haemostasis to occur.

During the inflammation stage, neutrophils enter the wound area to perform phagocytosis [9]. Within 48 h, macrophages enter the wound area and perform phagocytosis to debride the wound of pathogens [9, 10]. Once pathogens are eradicated, macrophage becomes the M2 subset, which releases TGFβ, PDGF, FGF and VEGF [10, 11, 12].

**FIGURE 1:**
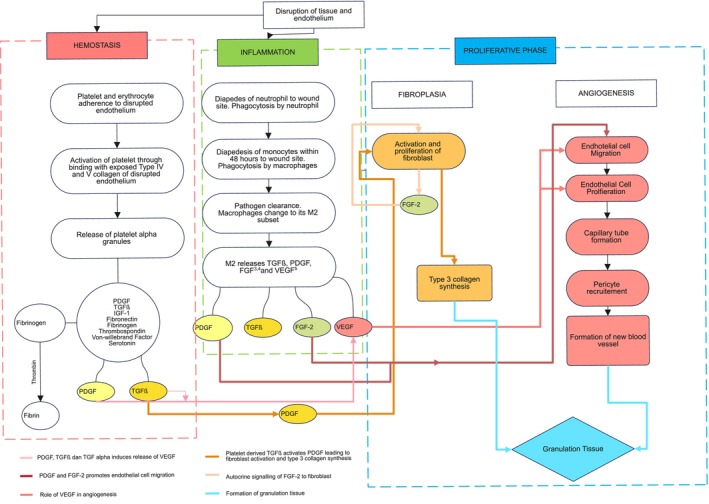
The wound healing process [5, 9, 10, 11, 12, 13]. The wound healing process consists of four overlapping phases: Haemostasis, inflammation, proliferative and remodelling. This figure focuses on the first three phases. The proliferative phase consists of fibroplasia and angiogenesis with granulation tissue as its outcome. Fibroplasia (activation of fibroblast) is affected by primarily PDGF and TGFβ as products of the haemostasis and inflammation phases. Angiogenesis is affected by PDGF, TGFβ and FGF‐2 as products of the previous phases. FGF‐2, fibroblast growth factor‐2; IGF‐1, insulin growth factor‐1; PDGF, platelet derived growth factor; TGFβ, transforming growth factor β; VEGF, vascular endothelial growth factor.

The result of the proliferative phase is the production of granulation tissue. Granulation tissue is produced by a synthesis of type III collagen by fibroblasts and the formation of new blood vessels through angiogenesis [10]. Products of haemostasis and inflammation contribute to the synthesis of collagen and angiogenesis. Focusing on PDGF and TGFβ, PDGF and TGF β released during the haemostasis and inflammation phase form VEGF that causes migration of endothelium to wound and proliferation of endothelial cells during the angiogenesis process [10]. PDGF and FGF‐2 released through the inflammation phase also aids in endothelial migration to wound [10, 13]. Fibroblasts are activated by a cascade of activation from platelet to TGFβ and PDGF. The activation of fibroblasts causes the synthesis of type 3 collagen [5]. The synthesis of type 3 collagen, along with angiogenesis, causes granulation tissue formation. (Figure 1).

Similar stages of haemostasis, inflammation and proliferation can be observed in mucosal or skin tissue. The main difference between the two is that mucosal wounds heal faster with less scarring than skin wounds due to stem cell levels, cellular proliferation capacity and intrinsic characteristics of epithelium [14].

### 2.2 PDGF

Platelet‐derived growth Factors (PDGFs) are a group of proteins that play a pivotal role in various wound repair stages, including granulation tissue formation. They were first discovered in the 1970s. Within this group, PDGFs manifest as individual protein monomers denoted as PDGF‐A, ‐B, ‐C and ‐D, which possess the capacity to undergo disulfide‐linked association, forming both homodimeric and heterodimeric combinations, such as PDGF‐AA, ‐BB, –AB, ‐CC and ‐DD. Platelets, fibroblasts and vascular endothelial cells excrete these proteins. Their significance lies in their potent mitogenic capabilities towards mesenchymal cells, mainly fibroblasts and smooth muscle cells, thus exerting a profound influence on wound healing processes, notably granulation tissue formation. This influence extends to fibroblast proliferation, mesenchymal stem cell (MSC) recruitment and the synthesis of extracellular matrix (ECM), thereby substantiating their pivotal role in wound healing cascades.

#### 2.2.1 PDGF Signalling Pathway

**FIGURE 2:**
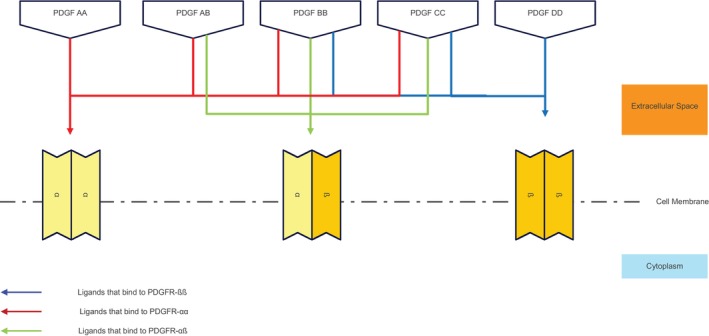
Interaction between PDGF ligands and PDGFR homodimers/heterodimers [1, 15, 16]. PDGF subunits form heterodimeric or homodimeric combinations PDGF‐AA, PDGF‐AB, PDGF‐BB, PDGF‐CC and PDGF‐DD. Two types of PDGF receptor exist: PDGFR‐α and PDGFR‐β. These receptors come in dimeric form PDGFR‐ αα, PDGFR‐ αβ and PDGFR‐ ββ. PDGF subunits A, B and C exhibit binding affinity towards PDGFR‐α, while subunits B, C, and D display affinity for PDGFR‐β. Consequently, PDGF‐AA selectively engages PDGFR‐αα receptors, whereas PDGF‐BB binds to both PDGFR‐αα and PDGFR‐αβ. Additionally, PDGF‐CC interacts with PDGFR‐αα, PDGFR‐αβ, and PDGFR‐ββ, whereas PDGF‐DD exclusively binds to PDGFR‐ββ. PDGF, platelet derived growth factor; PDGFR: platelet derived growth factor receptor.

PDGF receptors belong to the tyrosine kinase receptor class, a pivotal category in cellular signalling pathways [1]. Consisting of PDGFR‐α and PDGFR‐β monomers, these receptors transition into homodimers or heterodimers upon activation by PDGF ligands [1]. Notably, PDGF subunits A, B and C exhibit binding affinity towards PDGFR‐α, while subunits B, C and D display affinity for PDGFR‐β [1, 15, 16]. Consequently, PDGF‐AA selectively engages PDGFR‐αα receptors, whereas PDGF‐BB binds to both PDGFR‐αα and PDGFR‐αβ. Additionally, PDGF‐CC interacts with PDGFR‐αα, PDGFR‐αβ and PDGFR‐ββ, whereas PDGF‐DD exclusively binds to PDGFR‐ββ (see Table 1 and Figure 2) [1, 15, 16]. This intricate receptor‐ligand interaction delineates the specificity and diversity within the PDGF signalling network, underscoring its significance in cellular communication and physiological processes.

**TABLE 1:** PDGF ligand and interaction with PDGF receptors [1, 15, 16].

PDGF ligand	PDGF receptors
PDGF AA	Receptor αα
PDGF AB	Receptor αα
	Receptor αβ
PDGF BB	Receptor αα
	Receptor αβ
	Receptor ββ
PDGF CC	Receptor αα
	Receptor αβ
	Receptor ββ
PDGF DD	Receptor ββ

**FIGURE 3:**
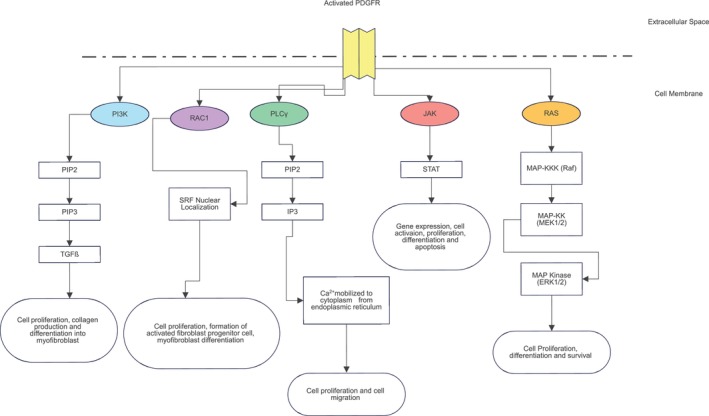
PDGF signalling cascade in fibroblasts [18, 19, 20, 21, 22]. Activation of PDGFR activates several pathways leading to cell proliferation, collagen production, myofibroblast differentiation and cell migration. IP3, inositol (1,4,5)‐triphosphate; MAP‐KK, MAP‐kinase kinase/MEK 1/2; MAP‐KKK, MAP‐kinase kinase kinase/RAF; PDGFR, platelet derived growth factor receptor; PIP‐2, phosphatydylinsoitol (4,5)‐biphosphate; PIP3, phosphatidylinositol (3,4,5)‐triphosphate; SRF, serum response factor; TGFβ, transforming growth factor β.

Activation of PDGFR, a tyrosine kinase receptor family member, initiates a cascade of intracellular signalling events. Upon activation, PDGFR triggers the Ras pathway and this prompts the Ras protein to become activated, subsequently leading to the activation of MAP Kinase kinase kinase (RAF). Once RAF is activated, it stimulates MAP Kinase kinase (MEK 1/2), activating MAP Kinase (ERK 1/2). The activation of ERK 1/2 results in a series of downstream effects, including alterations in protein activity and gene expression. This sequential activation of signalling molecules highlights the intricate and coordinated nature of cellular response pathways triggered by PDGFR activation (Figure 3) [17].

In the context of hepatic stellate cells (HSC), the activation of PDGFR is crucial in driving collagen synthesis and contributing to the progression of hepatic fibrosis. Upon PDGFR activation, the MAP kinase pathway, particularly ERK1/2, undergoes phosphorylation, leading to the modulation of gene expression directly from DNA. This modulation includes the upregulation of transcription factors such as activator protein 1 (AP‐1) and nuclear factor κB (NF‐κB). The expression of these transcription factors subsequently orchestrates the activation of target genes involved in collagen synthesis, including type 1 collagen, as well as regulators of matrix remodelling such as tissue inhibitors of metalloproteinases (TIMPs) and matrix metalloproteinases (MMPs). This cascade of events underscores the pivotal role of PDGFR‐mediated signalling in driving the fibrogenic response in hepatic stellate cells, thereby contributing to the pathogenesis of hepatic fibrosis [23].

PDGF binds to tyrosine kinase receptors on cell surfaces composed of PDGFR‐ α or PDGFR‐β, where dimerization occurs further, resulting in cross‐linking phosphorylation on the receptor of the cytoplasmic region. This then activates PDGF signalling pathways consisting of Inositol (1,4,5)‐triphosphate (PI3K), Rac1, Phospholipase C Gamma (PLCγ), Janus Kinase/Signal Transducers and Activators of Transcriptions (JAK/STAT) and RAS Pathway among many others to induce fibroblast proliferation, production, migration and differentiation. In an idiopathic pulmonary fibrosis (IPF) case, it was found that PI3K signal transduction enzyme catalyses PIP2 to PIP3, then further induces TGFβ for cell proliferation, collagen production and differentiation into myofibroblast, causing IPF [18]. Rac1, on the other hand, when inhibited, decreases cell migration ability and shows more formation of lamellipodium by upregulating serum response factor nuclear localization [22]. Another activated pathway is PLCγ; this receptor mediates the hydrolysis of PIP2 to IP3, leading to the mobilisation of calcium influx towards the cytoplasm from the endoplasmic reticulum. Calcium is vital in wound healing as it ensures extracellular and intracellular signalling pathways to forego for keratinocytes and fibroblasts [21]. Furthermore, JAK/STAT activates gene expression as well as cell activation, proliferation and differentiation [24]. Meanwhile, the RAS pathway involves a sequential phosphorylation event from MAP kinase kinase kinase (Raf) phosphorylating MAP kinase kinase (MEK 1/2), resulting in dual phosphorylation of MAP‐kinase (ERK 1/2) to induce cell proliferation, differentiation and survival. All these pathways, in turn, will enhance fibroblast proliferation, collagen production and differentiation (Figure 3).

#### 2.2.2 PDGF‐BB, PDGF‐AB and PDGF‐AA in Granulation Formation

PDGF‐AA, PDGF‐BB and PDGF‐AB were discovered in the 1970s, while PDGF‐CC and PDGF‐DD were discovered more recently, in the 1990s [25]. Among the various homo‐ and heterodimers of PDGF, PDGF‐BB, PDGF‐AB and PDGF‐AA emerge as pivotal players in the formation of granulation tissue. Notably, findings from an in vivo and in vitro investigation conducted by Lepistö et al. shed light on the differential effects of PDGF‐BB in collagen synthesis [26]. The in vivo study utilised implantation of viscose cellulose sponge cylinders injected with 5, 50 or 500 ng of PDGF‐BB. The study reported a significant increase in collagen synthesis, as evidenced by elevated accumulation of hydroxyproline concentrations, following the administration of 500 ng of PDGF‐BB in vivo. In the in vitro setting, fibroblast cultures started from rat granulation tissue were used and supplemented with ascorbic acid and 1, 10 or 30 ng PDGF‐BB. Rate of collagen synthesis was measured as synthesis of peptide bound hydroxyproline and calculated per cell. The in vitro study demonstrated that PDGF‐BB stimulated the proliferation of fibroblasts, and was also dose dependent indicating its role in promoting cellular growth. However the rate of hydroxyproline synthesis diminished per cell as the dose of PDGF‐BB became higher [26]. It can be inferred that PDGF‐BB induces collagen synthesis by means of fibroblast proliferation and not amount of collagen per cell. As numbers of fibroblast increase, so does the accumulation concentration of hydroxyproline.

Moreover, the investigation by Lepist et al. also utilised PDGF‐AA in several doses both in vitro and in vivo. The doses utilised were the same as PDGF‐BB. In the in vivo setting, it was revealed that no steady trend could be observed in the concentrations of hydroxyproline as higher doses of PDGF‐AA were given: There was a 2% increase of hydroxyproline concentration in comparison to control when 50 ng of PDGF‐AA was given however there was a 6% decrease when 500 ng of PDGF AA was given. Nevertheless, it exhibited a stimulatory effect on fibroblast proliferation in vitro, albeit without a concurrent increase in hydroxyproline levels [26].

A study by Gilbertson et al. supported this. The in vitro study utilises the insertion of cDNA into adenovirus that would express genes for PDGF and release PDGF proteins. From this study, it is concluded that PDGF‐BB and PDGF‐AB play a significant role in granulation tissue formation compared to PDGF‐AA [16]. A study by Jian et al. reported the role of PDGF‐BB in fibroblast proliferation and angiogenesis. Therefore, it can be concluded that PDGF‐BB and PDGF‐AB play a major role in granulation tissue formation [27].

### 2.3 TGFβ

The TGFβ is a multifunctional growth factor that is produced by platelets, keratinocytes, macrophages and fibroblasts. It is a crucial cytokine for preserving skin homeostasis. During wound healing, signalling of TGFβ controls reepithelization, inflammation, angiogenesis and the creation of granulation tissue. The control of TGFβ over keratinocyte migration and proliferation is how this growth factor highly attributes to wound healing. By controlling the keratinocyte cell cycle and preventing proliferation, TGFβ signalling helps maintain tissue homeostasis in unwounded skin. The role of TGFβ signalling in both healthy and compromised wound emphasises how it highly affects reepithelization [26, 27].

#### 2.3.1 TGFβ Signalling Pathway

**FIGURE 4:**
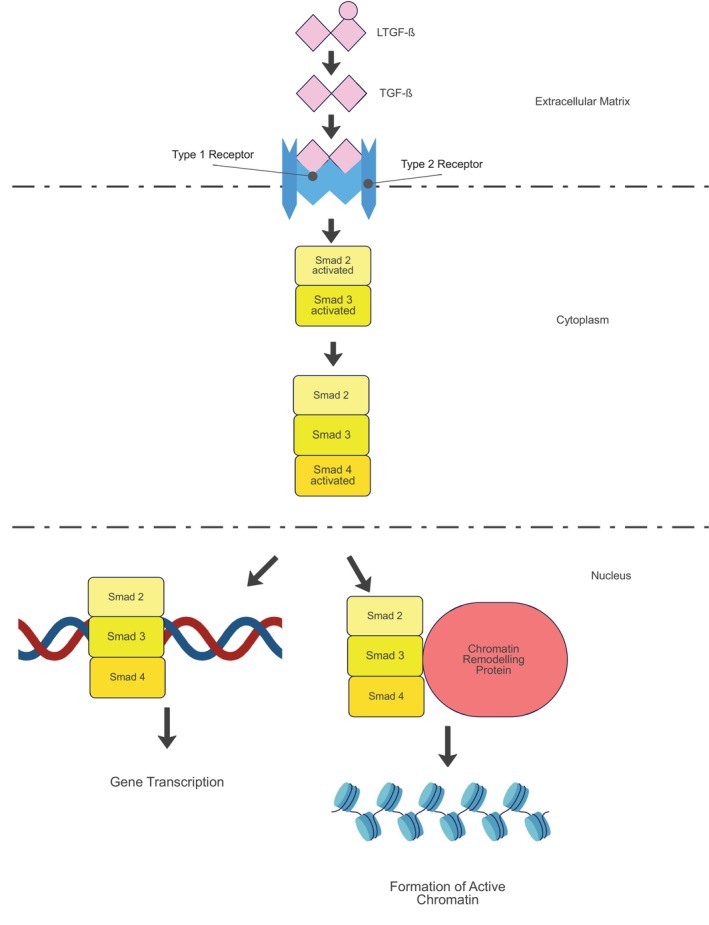
TGFβ R‐Smad signalling pathway [28, 29]. LTGFβ: latent transforming growth factor β, TGFβ: transforming growth factor β. LTGFβ is activated into TGFβ. TGFβ a dimer kinase binds to Type 1 and Type 2 receptors. Once activated these receptors activate Smad proteins 2 and 3. Smad 2 and 3 then activate Smad 4 and form a complex. This protein complex translocates to the nucleus to perform gene transcription and form active chromatin. This model summarises explanation from Lichtman et al. and Demidova‐Rice et al.

TGFβ is one of the most influential growth factors in granulation tissue formation. There are three TGFβ isoforms: TGFβ‐1, TGFβ‐2 and TGFβ‐3. The TGF‐β isoforms are secreted as latent forms in a dimerized complex (LTGFβ) within the extracellular matrix. This dual‐specificity kinase will form a heterotetrameric receptor complex with two type I receptors, and two type II receptors. Once it is bound, a cytoplasmic kinase domain of type I receptor signalling is transmitted. This will then phosphorylate receptor‐regulated SMAD proteins, upregulating the SMAD pathway. Another activated pathway is the non‐SMAD signalling pathway through types I, II receptors and interacting proteins. Activated R‐SMAD proteins (SMAD 2 and SMAD 3) form a complex with the common‐SMAD, SMAD4. It then translocates to the nucleus to regulate transcription of target genes together with cofactors. R‐SMADS also form a complex with chromatin remodelling protein that promotes formation of active chromatin. (Figure 4) This is a prerequisite for transcriptional activation by R‐SMAD complexes. R‐SMAD proteins also regulate biogenesis of primary miRNA transcripts by processing by Drosha microprocessor complex. All isoforms of TGFβ: TGFβ‐1, TGFβ‐2 and TGFβ‐3 bind and compete to the same receptors [28]. However, each TGFβ isoform expresses different outcomes in vivo and has different wound healing functions. TGFβ‐1 plays a crucial role in granulation tissue and fibrosis formation, whereas TGFβ‐3 may have antifibrotic properties [28, 29].

#### 2.3.2 TGFβ in Granulation Tissue Formation

**FIGURE 5:**
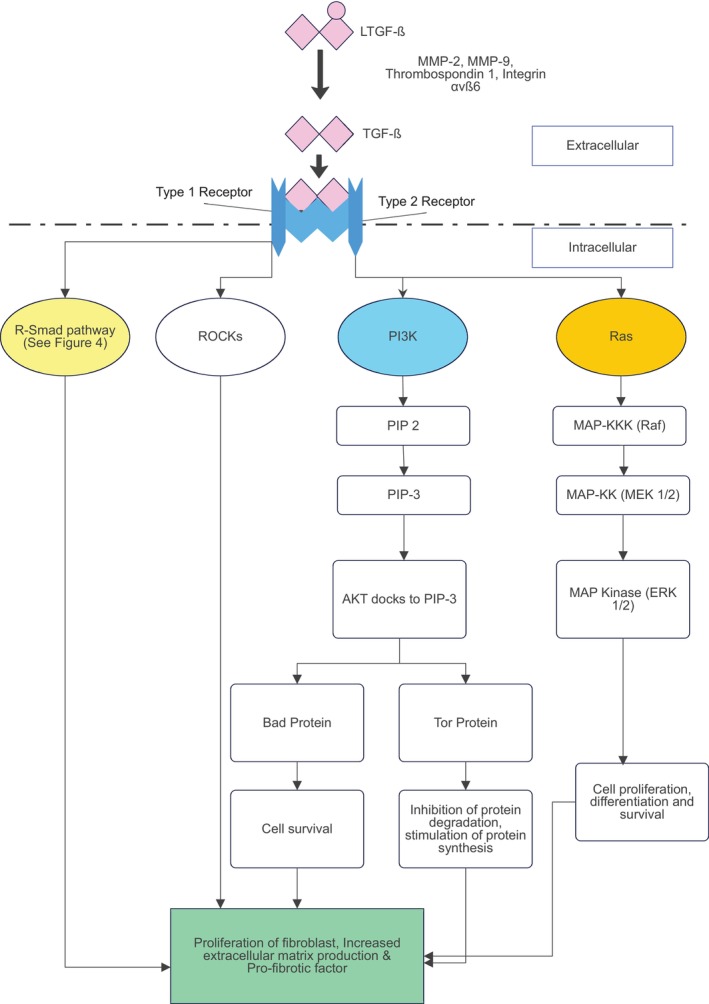
TGFβ signalling in fibroblast [17, 29]. After activation of receptors by TGFβ, R‐SMAD pathway, Rho‐associated coiled‐coil containing ROCKs, RAS–ERK pathway and PI3/AKT pathway are activated. This leads to the regulation of myofibroblast generation, fibroblast proliferation/ apoptosis, extracellular matrix (ECM) production and also upregulates DNA transcription factors, co‐activators and co‐repressors to regulate gene expression. LTGFβ, latent transforming growth factor β; MAP‐KK, MAP‐kinase kinase/MEK 1/2; MAP‐KKK, MAP‐kinase kinase kinase/RAF; PIP‐2, phosphatidylinositol (4,5)‐biphosphate; PIP3, phosphatidylinositol (3,4,5)‐triphosphate; ROCKs, Rho‐associated coiled‐coil containing protein kinases; TGFβ, transforming growth factor β.

The TGFβ family is produced by platelets, keratinocytes, macrophages and fibroblasts. There are three isomers of TGFβ which includes: TGFβ1, 2 and 3. Generally latent TGFβ is stored in the extracellular matrix in a dimerized latent complex, which is stored until activated. As mentioned previously, LTGFβ is activated by serum proteases. These serum proteases include MMP‐2, MMP‐9, Thrombospondin 1 and integrin αvβ6. The TGFβ binds to TβRII, a serine/threonine kinase receptor which, when activated, recruits and phosphorylates TβRI along with other various intracellular signalling cascades. After activation of both receptors, R‐SMAD pathway, Rho‐associated coiled‐coil containing protein kinases (ROCKs), MAP Kinase (ERK) and PI3/AKT are activated. Thus leading to the regulation of myofibroblast generation, fibroblast proliferation/ apoptosis, extracellular matrix (ECM) production and also upregulates DNA transcription factors, co‐activators and co‐repressors to regulate gene expression. The upregulated gene expression then results in increased myofibroblast generation, elevated ECM production and pro‐fibrotic factor connective tissue growth factor (CTGF) to promote fibrosis, which eventually leads to granulation tissue formation (Figure 5) [29].

### 2.4 Role of PDGF and TGFβ in Excessive Granulation

Excessive granulation is an abnormal wound‐healing process. Both PDGF and TGFβ are involved in this process alongside other growth factors such as VEGF and FGF. However PDGF and TGFβ are the main two significant factors and there are literatures that describe interaction between the two. It was found that PDGF can enhance TGFβ signalling by promoting activation of SMAD proteins, which are crucial for TGFβ's effect on cells, as well as to modulate each other's signalling and stability [7, 30]. Elevated expression of PDGF underlies the pathogenesis of hypertrophic scar and keloids due to increased fibroblast proliferation and extracellular matrix [31]. Therefore, it can be inferred that an increase in fibroblast proliferation and extracellular matrix deposition of collagen, fibronectin and proteoglycans can also give rise to excessive granulation tissue. PDGF‐AA and ‐BB were shown to increase the number of fibroblasts from granulation tissue, and these cells are responsible for synthesising ECM components. PDGFs also have MMP inhibition activity and helps increase cellular recruitment to inflammatory sites. An in vivo study using subcutaneously implanted cellulose sponges in rats showed an 34% increase in collagen accumulation when 500 ng of PDGF‐BB from yeast cell transfected were given to the rats, whilst also showing enhanced cellular activity and ECM synthesis. Additionally TGFβ can also inhibit matrix metalloproteinase (MMPs) which are responsible for degrading ECM components which further exacerbates ECM accumulation [26, 32]. The increment of PDGF action may be due to increased expression of PDGF receptors in cells, including fibroblasts resulting from injury and the presence of exogenous growth factors such as EGF [29]. At the same time, elevated TGFβ also leads to excessive granulation through upregulated fibroblast activation and myofibroblast differentiation, increased ECM production and deposition while inhibiting MMPs, inflammatory response modulation and its interaction with PDGF. Both these processes result in overabundance of granulation tissue, which can impede normal healing and lead to excessive granulation tissue formation.

A previous study involved a group of genetically modified mice with higher systemic TGFβ‐1 levels and mice with normal TGFβ‐1 levels involved. Wounds were created on the dorsum of both groups of mice, and subcutaneous implantation of a Polyvinyl Alcohol (PVA) sponge was performed. After follow‐up, PVA sponges of mice with higher systemic TGFβ‐1 levels showed increased cellularity, granulation tissue formation and collagen deposition [31]. Some studies have shown that inhibition of TGFβ in chronic wounds have demonstrated inhibition in keratinocytes of nonhealing venous ulcers thus showing a decreased expression of TGFβ receptors, loss of Smad2 and deregulation of TGFβ target genes. This then leads to loss of tissue homeostasis and inability of keratinocytes to migrate and close a wound. However inhibition of TGFβ and PDGF in acute wounds can be potential research topics in aucte wounds [33].

### 2.5 Clinical Implications of Excessive Granulation Tissue in the Opthalmology Field: Excessive Granulation Tissue and Endo‐DCR Outcomes

Excessive granulation tissue formation may disrupt the postoperative wound healing process, resulting in various complications and unresolved cases. In the ophthalmology field, excessive granulation tissue hinders outcomes of endo‐dacryocystorhinostomy (Endo‐DCR) procedures performed in nasolacrimal duct obstruction patients. This procedure creates an ostium adjacent to the lacrimal sac and incorporates the lacrimal sac with the lateral nasal mucosa to bypass the nasolacrimal duct obstruction [34]. However, if granulation tissue formation occurs in the ostium (may be referred to as ostium granulation), this may result in anatomical failure of the procedure and may lead to ostium stenosis.

It was hypothesized that a secondary intention closure of wound healing may have caused ostium granulation due to mucosal resection and excessive inflammation that occurs chronically within the nasal mucosa, with factors such as pollutants or cigarette smoke as additional factors causing chronic inflammation [10, 35]. To prevent the formation of ostium granulation, steroid and mitomycin C (MMC) have been previously used in endo‐DCR procedures.

Topical steroid agents such as intraoperative nasal packing of triamcinolone acetate 40 mg or budesonide nasal spray four times a day for 4 months after surgery is known to inhibit mRNA expression of TGFβ and thus can reduce the formation of ostium granulation, decrease pain and accelerate reepithelization [36, 37].

MMC, on the other hand, is an antineoplastic drug that inhibits DNA synthesis and proliferation of fibroblast cells, thus reducing collagen synthesis and vascular ingrowth, showing an ostium patency rate of 95.5% in the MMC group compared with 88.6% in the conventional group. Numerous side effects, however, have been documented by the usage of MMC, such as severe secondary glaucoma, secondary cataract and scleral calcification during pterygium surgery or hypotony‐related maculopathy, infection and endophthalmitis in glaucoma filtration surgery [38, 39].

Despite employing various techniques aimed at preventing ostium granulation, such as preserving mucosal flaps to minimise secondary wound healing and administering intranasal topical corticosteroids to reduce chronic inflammation, the occurrence of ostium granulation remains a challenge. Furthermore, Mitomycin‐C (MMC), a potent agent commonly used to mitigate granulation tissue formation, may be limited in specific medical centers. This highlights the ongoing need for alternative strategies or therapies to effectively manage ostium granulation, particularly in cases where MMC is not readily accessible.

This then raises the question of whether medications that work on PDGF receptor blockers could be useful in reducing excessive granulation formation during wound healing. Olaratumab is a human monoclonal antibody against PDGF‐ α, thus hindering the interaction between PDGF‐AA and PDGF‐BB ligands towards PDGFR‐αα and PDGFR‐αβ receptors, thus reducing the autophosphorylation process.

Olaratumab is also a targeted therapy of high affinity towards PDGFR‐ α, thus ensuring that other enzyme kinase‐linked receptors would be minimally affected, but due to its interaction with PDGFR‐ α, mitogen‐activated protein kinases (MAPKs) and Protein Kinase B (PKB, or Akt) pathway activity will also be reduced. Other effects are inhibiting mitogenesis and upregulating endocytosis of PDGFR, thus reducing the amount of PDGF receptors that bind to PDGF ligands [40, 41, 42]. Although Olaratumab seems promising to reduce excessive granulation tissue, Olaratumab is currently used for sarcomas and carcinomas [40]. The mechanism of action involved for cancer calls is through by binding PDGFR‐ α and disrupting tumour cell proliferation, survival, migration and reducing tumour growth and spread by downstreaming signalling cascades and kinases like Akt and MAPK. Thus, further research is still needed to ensure that Olaratumab can have this effect on the wound‐healing process.

## 3 Conclusion

PDGF and TGFβ are critical growth factors involved to the formulation of granulation tissue formation, playing significant roles in normal wound healing as well as in pathological conditions like excessive granulation. PDGF and TGFβ are expressed during haemostasis and inflammatory phase of wound healing and aid in fibroblast activation and angiogenesis through activation of VEGF or direct effect on endothelial cell migration. These two actions result in type III collagen synthesis and new blood vessel formation, thus the formation of granulation tissue. PDGF activate signalling pathways to promote fibroblast proliferation, ECM matrix synthesis and angiogenesis which are essential steps for granulation tissue development. Similarly, TGFβ, through its SMAD and non‐SMAD signalling pathway, controls fibroblast proliferation, myofibroblast differentiation and ECM deposition. If dysregulation occurs during this process, marked by elevated PDGF and TGFβ, this can lead to excessive granulation tissue from an overactivated fibroblast activity and increased ECM deposition as seen in cases of hypertrophic scars, keloids or post‐operative complications in ophthalmology procedures like Endo‐DCR.

## Author Contributions

**Josiah Irma:** conceptualization (lead), project administration (lead), visualization (lead), writing – original draft (lead), writing – review and editing (lead). **Arief S. Kartasasmita:** conceptualization (lead), supervision (lead), writing – review and editing (supporting). **Angga Kartiwa:** conceptualization (lead), supervision (lead), writing – review and editing (supporting). **Irawati Irfani:** conceptualization (lead), supervision (lead), writing – review and editing (supporting). **Saraswati Anindita Rizki:** conceptualization (supporting), visualization (supporting), writing – original draft (supporting), writing – review and editing (supporting). **Serena Onasis:** conceptualization (supporting), visualization (supporting), writing – original draft (supporting), writing – review and editing (supporting).

## Ethics Statement

The authors have nothing to report.

## Consent

The authors have nothing to report.

## Conflicts of Interest

The authors declare no conflicts of interest.

## Permission to Reproduce Materials

This article includes excerpts from previously published works, proper attribution is provided in the figure as well as in‐text citations. Any materials reproduced in this review adheres to the terms and conditions of original publishers.

## Data Availability

Data sharing not applicable to this article as no datasets were generated or analysed during the current study.
